# Component Microenvironments and System Biogeography Structure Microorganism Distributions in Recirculating Aquaculture and Aquaponic Systems

**DOI:** 10.1128/mSphere.00143-19

**Published:** 2019-07-03

**Authors:** Ryan P. Bartelme, Matthew C. Smith, Osvaldo J. Sepulveda-Villet, Ryan J. Newton

**Affiliations:** a School of Freshwater Sciences, University of Wisconsin—Milwaukee, Milwaukee, Wisconsin, USA; National Institute of Advanced Industrial Science and Technology

**Keywords:** *Flavobacterium*, *Nitrospira*, ammonia-oxidizing *Archaea*, aquaponics, biofilter, comammox, microbial community composition, nitrifiers, recirculating aquaculture system

## Abstract

Flowthrough and pond aquaculture system microbiome management practices aim to mitigate fish disease and stress. However, the operational success of recirculating aquaculture systems (RAS) depends directly on system microbial community activities. In RAS, each component environment is engineered for a specific microbial niche for waste management, as the water continuously flowing through the system must be processed before returning to the rearing tank. In this study, we compared waste management component microbiomes (rearing tank water, pH correction tank, solid-waste clarifier, biofilter, and degassing tower) within a commercial-scale freshwater RAS by high-throughput 16S rRNA gene sequencing. To assess consistency among freshwater RAS microbiomes, we also compared the microbial community compositions of six aquaculture and aquaponic farms. Community assemblages reflected site and source water relationships, and the presence of a hydroponic subsystem was a major community determinant. In contrast to the facility-specific community composition, some sequence variants, mainly classified into *Flavobacterium*, *Cetobacterium*, the family *Sphingomonadaceae*, and nitrifying guilds of ammonia-oxidizing archaea and *Nitrospira*, were common across all facilities. The findings of this study suggest that, independently of system design, core taxa exist across RAS rearing similar fish species but that system design informs the individual aquatic microbiome assemblages. Future RAS design would benefit from understanding the roles of these core taxa and then capitalizing on their activities to further reduce system waste/added operational controls.

**IMPORTANCE** Recirculating aquaculture systems (RAS) are agroecosystems for intensive on-land cultivation of products of fisheries. Practitioners that incorporate edible plant production into RAS refer to these facilities as aquaponic systems (AP). RAS have the potential to offset declining production levels of wild global fisheries while reducing waste and product distance to market, but system optimization is needed to reduce costs. Both RAS and AP rely on microbial consortia for maintaining water quality and promoting fish/plant health, but little is known about the microorganisms actually present. This lack of knowledge prevents optimization of designs and operational controls to target the growth of beneficial microbial species or consortia. The significance of our research is in identifying the common microorganisms that inhabit production RAS and AP and the operational factors that influence which microorganisms colonize and become abundant. Identifying these organisms is a first step toward advanced control of microbial activities that improve reproducibility and reduce costs.

## Abstract (precis)

Recirculating aquaculture systems (RAS) are agroecosystems for intensive on-land cultivation of products of fisheries. Practitioners that incorporate edible plant production into RAS refer to these facilities as aquaponic systems (AP). RAS have the potential to offset declining production levels of wild global fisheries while reducing waste and product distance to market, but system optimization is needed to reduce costs. Both RAS and AP rely on microbial consortia for maintaining water quality and promoting fish/plant health, but little is known about the microorganisms actually present. This lack of knowledge prevents optimization of designs and operational controls to target the growth of beneficial microbial species or consortia. The significance of our research is in identifying the common microorganisms that inhabit production RAS and AP and the operational factors that influence which microorganisms colonize and become abundant. Identifying these organisms is a first step toward advanced control of microbial activities that improve reproducibility and reduce costs.

## INTRODUCTION

Aquaculture is the cultivation of products of fisheries for human use or consumption. Early system designs consisted of ponds, pens, and continuous water flowthrough setups for cultivating finfish or other aquatic foods. Now, practices also include highly engineered recirculating aquaculture systems (RAS). These systems are constructed to optimize water use, often achieving a 90% to 99% reduction in water consumption compared to more-conventional methods (1). Nevertheless, recirculating water results in decreased water quality, primarily through the accumulation of fish waste and uneaten food (2). RAS typically manage water quality by implementing components to capture and remove solid and nitrogenous waste products before returning water to the production tank (3, 4). RAS component process flow typically progresses as follows: water from the production tank (the tank for fish grow-out) is sent to a device designed for removal of particulate solids (e.g., a settling tank or clarifier), then to a nitrifying biofilter to remove ammonia, and finally to components that further clean and chemically condition the water (e.g., degasser, ozone generator or UV light generator, oxygenator). The reconditioned water is then returned to the production tank. This internal waste-recycling setup means that RAS offer a potentially long-term sustainable offset for the declining productivity of capture fisheries (5). However, RAS success rates must grow if they are to be part of a solution to meet increased demand for products of global fisheries by increasing global supply (6).

In the 1970s, aquacultural engineers supplemented nitrifying biofilters with plants for secondary treatment of nitrogenous waste (7–9). Today, such systems are commonly called aquaponic systems (AP), a portmanteau of aquaculture and hydroponics, where hydroponic subsystems are added to a RAS downstream of the solids removal and nitrifying biofilter components. Aquaponic systems tend to be profitable only when operated with a plant-centered production schedule (10) and therefore do not offer the same benefits as RAS for offsetting declining production levels of capture fisheries. Soilless systems, however, offer several advantages compared to traditional soil agriculture, such as lower energy and water footprints (11), a grow season unbound by climate, and greater control over pest management (12). Both RAS and aquaponic systems also have the ability to reduce the product distance to market (10, 13). However, neither RAS nor aquaponic systems dominate controlled environmental agriculture due to the start-up phase being both financially demanding and knowledge intensive (14). The nitrifying biofilter is often considered the most important microbial component of a RAS, as without its conversion of ammonia to nitrate, ammonia levels accumulate quickly to concentrations that are toxic to fish (3). For this reason, the nitrifying biofilter must be established before production can begin. Often, start-up begins by flowing water through the system while dosing with ammonia or adding low levels of fish as the ammonia source. During this period, nitrifying microorganisms grow to high densities in the biofilter but usually require several weeks of growth before ammonia levels are safe for production (1).

Since RAS and aquaponic systems are engineered for biological output, their success depends in part on operationally controlling microbial activities. These controls are complex. Both approaches rely on a diverse consortium of microorganisms to carry out waste removal but also must govern production organism (plant and/or animal) microbiota interactions (pathogens and commensals). The multifaceted roles of microorganisms in RAS/aquaponic success have garnered some recent research attention. For example, fish have been found to be very sensitive to the external microbiome (15). In RAS, tank water microbiota composition was correlated with improved larval fish survival (16), and fish gut microbiota had high levels of taxonomic overlap with the plant root rhizosphere (17). Microorganisms also likely remineralize nutrients to support plant growth in aquaponic systems (18). These results highlight that, continuous water flow connects individual component microbiomes in both RAS and aquaponic systems, and thus microbial community assembly in each component’s microbiome may influence a separate component, including fish and/or plant health (15, 19). Also, the engineered nature of RAS may alter typical relationships between hosts and their microbiota, as significant differences in gut microbial composition between farm raised and wild fish have been noted previously (20). Although advances have been made, understanding of the microbial communities and activities in RAS/aquaponic systems lags far behind that of industries employing similarly engineered microbial processes such as municipal wastewater engineering and traditional soil crop agriculture (19). In part, this lag stems from a lack in understanding of the microbial players in these systems, especially as related to component/operational influences on microbial community assembly and whether community assemblages are consistent across facilities.

While we do not claim that studying microbial communities in RAS and aquaponic systems will assuredly reduce cost, we believe that understanding microbial control points, which begins by identifying key microorganisms, will assist in lowering the knowledge costs associated with starting and operating these systems. In this study, we investigated the microbial community compositional correlations within a RAS’s components over a short time course and among six geographically separate freshwater RAS. To compare bacterial communities within a system, we examined a RAS at the University of Wisconsin–Milwaukee (UWM) School of Freshwater Sciences (SFS), which is equivalent to a medium-scale commercial system. The microbial communities in this system were then compared to those in two other RAS, three aquaponic systems, and a commercial-sized recirculating freshwater aquarium. From these comparisons, we sought to identify microbes that are common across systems and those that distinguish system component communities. Additionally, we investigated the influences of plant presence (i.e., aquaponic system) and source water on bacterial community composition. Since nitrifying guilds are critical to both RAS and aquaponic system success, we examined nitrifier assemblages in detail across all systems studied.

## RESULTS AND DISCUSSION

### RAS microenvironments harbor distinct microbial communities.

Two ecological diversity metrics (alpha diversity and beta diversity) were calculated to evaluate the existence of RAS component microenvironments and their effects on microbial community composition. Mean Shannon-Weaver index values and Pielou’s evenness values were similar across the individual UWM RAS components (Table 1), but when samples were aggregated by sample class, there were significant differences between the three primary habitats present (namely, planktonic water [Shannon-Weaver index values of 4.12 ± 0.54], clarifier sludge [4.27 ± 0.21], and biofilter biofilm [4.66 ± 0.20]; Kruksal-Wallis rank-sum test of sample class χ^2^ = 8.092, df = 2, *P* = 0.0175) (Table 1). The presence of ozonation incorporated into the UWM RAS is a possible explanation for the lower alpha diversity in the water samples, as the ozone system directly treats the water. Also, a previous study found that ozonation increases the taxonomic diversity of *Bacteroidetes* and *Proteobacteria* in RAS biofilms (21) and thus may be a driver of diversity differences between the planktonic and biofilm communities. The influence of ozonation on the planktonic microbial communities in these systems warrants further study. These results also support the hypothesis that planktonic microbial assemblages differ significantly from biofilm communities that form in sludge digestion or within the biofilter (22, 23). Rearing tank diversity (Shannon-Weaver index; *H*′ = 4.13 ± 0.13) was approximately 1.6× greater than that seen in a previous study of rearing tank alpha diversity assessed by denaturing gradient gel electrophoresis (DGGE) (*H*′ = 2.6 ± 0.09), while the former study and our data indicated similar levels of community evenness (Pielou’s *J *=* *0.66 ± 0.02 and *J = *0.64 ± 0.09, respectively; 16). Our higher alpha diversity values likely indicate that massively parallel sequencing better captures co-occurring populations in recirculating aquaculture systems than molecular fingerprinting methods such as DGGE.

**TABLE 1:** Alpha-diversity metrics across UWM RAS sites

Site^*a*^	*H*' ± SD^*b*^	*J* ± SD^*c*^	*n*^*d*^	Sample category^*e*^
Biofilter effluent	4.22 ± 0.32	0.68 ± 0.05	4	Plankton
Biofilter sand	4.66 ± 0.20	0.75 ± 0.04	6	Biofilm
Biofilter water	4.26 ± 0.74	0.68 ± 0.12	4	Plankton
Clarifier	4.27 ± 0.21	0.68 ± 0.03	6	Sludge
Clarifier effluent	4.38 ± 0.35	0.70 ± 0.06	4	Plankton
Degasser	4.20 ± 0.31	0.67 ± 0.05	5	Plankton
pH buffer tank	3.54 ± 1.41	0.57 ± 0.22	3	Plankton
Rearing tank	4.13 ± 0.13	0.66 ± 0.02	4	Plankton

a All alpha-diversity metrics were calculated across the University of Wisconsin—Milwaukee Recirculating Aquaculture System sites using the V6 16S rRNA gene sequence data set.

b Data represent Shannon-Weaver diversity index (*H’*) values. Standard deviation (SD) values are indicated after the mean values.

c Data represent Pielou’s evenness metric (*J*) values. Standard deviation (SD) values are indicated after the mean values.

d The number of samples at each site is indicated.

e Sample categories used in Kruskall-Wallis rank sum hypothesis testing are indicated.

**FIG 1:**
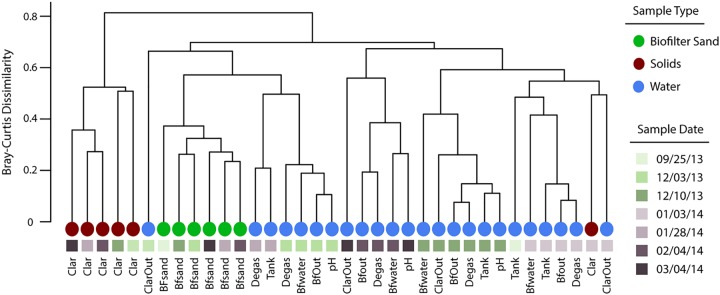
Dendrogram of bacterial community dissimilarity across University of Wisconsin–Milwaukee RAS components. The dendrogram was created using average-linkage Bray-Curtis dissimilarity of bacterial communities between RAS components. Leaves are labeled by date and sample site within the RAS. Sample site references are as follows, in order of process flow: Tank, rearing tank; pH, pH buffering tank; Clar, clarifier sludge; ClarOut, clarifier effluent; Bfsand, biofilter sand substrate, BfOut, biofilter effluent; Degas, carbon dioxide degassing tower.

Beta diversity was used to test whether each RAS component represented a unique microenvironment. The bacterial community composition clustered approximately by component (Fig. 1) (27.6% of the Bray-Curtis beta diversity was explained by component association alone [ADONIS; df = 4, *P* = 0.001]). The most discordant environment linkages resulted from either (i) samples classified as sludge that were more similar to chronologically linked nonsludge samples than to other sludge samples or (ii) a division in linkage patterns between water samples, where some samples were more closely associated to the sand biofilm samples than the remaining water samples (Fig. 1). Since all RAS components are connected by water flow, any single sampling period could reflect a relatively high level of release of microbes from one component (e.g., tank, biofilter, or digester) into the others. This action would result in homogenization of the community composition across components and explain some of our observed patterns. Additionally, changes in operator conditions during a rearing cycle can influence RAS microbial communities (22). These temporally punctuated whole-system changes may act to homogenize briefly communities across system components, but the major component types (water, sludge, and biofilter biofilms) generally harbor distinct microbial communities. RAS operation should include distinct management strategies for each component, while understanding that the flow of microorganisms between components has the potential, at least temporally, to alter the microbial activities in connected components.

### Cross-system comparison.

**FIG 2:**
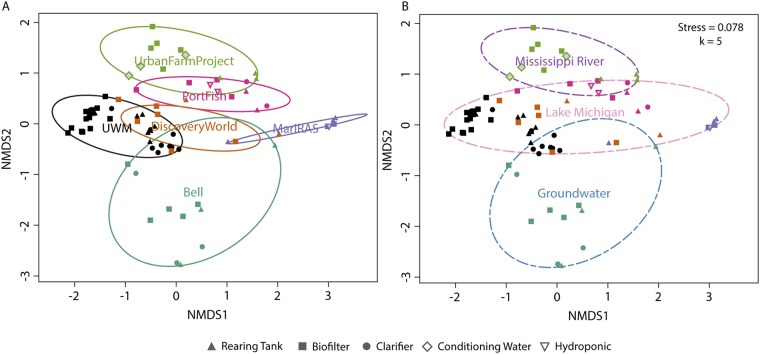
Nonmetric multidimensional scaling (nMDS) plot of bacterial community composition. The plot indicates Bray-Curtis dissimilarity between samples (V4-V5 16S rRNA gene data) as ordinated by the metaMDS function with dimensions *k* = 5 and stress = 0.078. Data in ellipses are illustrated as follows: (A) all samples from a particular site (ADONIS *R*^2^ = 0.355, *P* ≤ 0.001) and (B) all samples from a particular water source (ADONIS *R*^2^ = 0.110, *P* ≤ 0.001). Ellipses were added with the ordiellipse function in vegan (70). The component origin of each sample is indicated via the shape of the sample point as indicated in the figure key. UWM, University of Wisconsin—Milwaukee.

We found that both system site (i.e., individual facility) (envfit, vegan; *R*^2^ = 0.6491, *P* = 0.001) and water source (envfit, vegan; *R*^2^ = 0.2179, *P* = 0.001) correlated with bacterial beta diversity (nonmetric multidimensional scaling [nMDS] from Bray-Curtis dissimilarity with *k* = 5 dimensions and stress equal to 0.078; Fig. 2). The data representing the system site, as the dominating factor related to community composition, indicate that the conditions in each facility dictate strongly the resultant community assemblages. This “island biogeography”-like effect on community composition has been reported previously in aquaculture for individual tanks rearing the same fish species in a single facility (24). The biogeography could be due to differences in water conditions and nutrient concentrations in each facility, to random variation, or to a priority effect from the start-up phase, but ultimately, facility conditions dictate community assembly behavior more than component class (Fig. 2).

While beta diversity data among systems correlated less strongly with categorical factors such as system scale (envfit, vegan; *R*^2^ = 0.2016, *P* = 0.001), component class (envfit, vegan; *R*^2^ = 0.1935, *P* = 0.001), and aquaponics system (binary TRUE/FALSE; envfit, vegan; *R*^2^ = 0.1510, *P* = 0.001), these factors also influenced community composition. Although the facilities all operate differently and have unique community compositions, it is intriguing that source water could influence RAS/aquaponic system microbiomes (Fig. 2). The hypothesis that source water guides microbial community assembly is worth exploration, as this could be a critical and underexplored aspect of both RAS and aquaponic system design. Together, these data indicate that uniqueness in facility design and corresponding source materials results in facility-specific microbial assemblages, with different microbes presumably fulfilling similar niches across systems. However, some design features (e.g., a nitrifying biofilter, the presence of plants) modulate the bulk water community in predictable ways. In our study, all facilities, except Discovery World, reared yellow perch (*Perca flavescens*), so fish-specific microbiota relationships should have had minimal impact on the results. In a recent study, it was found that microbial community composition is dictated in part by system design, in this case, aquaculture versus coupled and decoupled aquaponic systems (25). We anticipate that further system design and operation standardization would result in microbial community assemblages that are more similar and thus more predictable among facilities.

**FIG 3:**
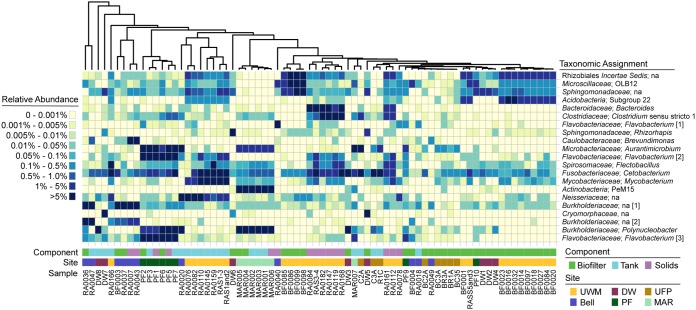
Heat map of the most abundant 16S rRNA gene amplicon sequence variants across component types. The plot indicates bacterial community composition relative to abundance data ordered by distance-based clustering (Euclidian) of samples containing only the illustrated amplicon sequence variants (ASVs). The top 10 most abundant ASVs (mean relative abundances) for each major component type, biofilter, solids, and rearing tank are plotted (heatmap2 from the ggplots R package). The ASVs are also ordered by distance-based clustering (Euclidian) of their relative abundance patterns. Gene relative abundances are indicated via color-coding as listed in the plot key. Each sample’s component type and sample site origin are indicated below the heat map. The sample name abbreviations are as follows: Bell, Bell Aquaculture; DW, Discovery World; PF, PortFish; UFP, UrbanFarmProject; MAR, Marinette. Family-level and genus-level taxonomic assignments are listed for each ASV. When these levels were not assigned, then the most refined taxonomic level is provided. Identical taxonomic assignments are given a number (indicated in square brackets) to facilitate references in the text. na, unassigned taxonomy at this level.

In contrast to the significant microbial community differences among facilities, some taxa (represented by unique amplicon sequence variants [ASVs]) were abundant in all samples (Fig. 3), showing that some taxa were maintained across all of the systems investigated. It is likely that these high abundance and ubiquitous microorganisms are continuously circulating through all components within RASs and represent planktonic communities that are generally associated with fish or fish feed. For example, a single ASV, identified as a potential *Cetobacterium* sp., was present at high abundance (1% to 5%) in all but 5 of the rearing tank and solids clarifier samples. *Cetobacterium* spp. are often found in freshwater fish intestinal tracts (26) and, in one study, occupied >75% of the fish fecal microbiome (27). A number of ASVs associated with the order *Rhizobiales* were also present in high relative abundances across RAS rearing tanks and biofilters but could not be taxonomically classified deeper than the order level. *Rhizobiales* spp. are also common fish intestinal microbes (28). Certainly, fish intestinal microbiota are dominant in RAS planktonic microbial communities and possibly act to homogenize assemblages across systems. Further information from studies examining multiple fish species reared in RAS or aquaponic systems is needed to determine whether the fish species alter the microorganisms present in RAS or if RAS conditions override natural fish intestine-microbiota associations.

Some probable non-host-associated microorganisms were also ubiquitous across RAS. A *Sphingomonadaceae* sequence (closest cultured relative, *Sphingorhabdus* sp. WM51; blastn, 100% query coverage; e = 0.0; 100% identity) was present at relative abundances of >0.001% in all but 12 of the 74 samples (Fig. 3). These results were consistent with what was reported previously in the UWM SFS RAS (22). Interestingly, these sphingomonads could be a boon for aquaculturalists, as some species actively and cooperatively degrade geosmin (29), which produces off-flavors in fish (30). Additionally, grown in coculture with a species of *Pseudomonas*, a sphingomonad was also shown to degrade 2-methylisoborneol (MEB) (31). MEB is another off-flavor-producing compound in aquaculture systems. Together, these data indicate there is untapped potential to optimize microorganism activity to improve production processes.

Most research on solids clarification in RAS and aquaponics focuses on a reduction of dissolved organic matter (32) and capture of solids to maintain nitrification rates in the biological filter (33, 34). The heterotrophic bacterial communities recovered from the biological filters in our study resembled those found previously (22, 35). Examples of abundant taxa shared with these previous biofilter studies include uncultured *Acidobacteria* and uncultured *Rhizobiales* as well as *Flavobacterium* spp. According to recent reviews, solids management is critical to controlling populations of heterotrophic bacteria, some of which may be opportunistic pathogens (23, 36). ASVs classified as *Flavobacterium* spp. were especially prevalent across all systems. Each of two different sequences of an unclassified *Flavobacterium* spp. (indicated by “[1]” and “[2]” in Fig. 3) was represented in either the solids or rearing tank samples, respectively, while a third *Flavobacterium* sequence (“[3]”; Fig. 3) was present in both. Our data suggest that *Flavobacterium* spp. may proliferate in the solids capture systems, presumably by exploiting the abundant sources of complex organic carbon (37). We also found that the V6 region of the 16S rRNA gene differentiated Flavobacterium columnare from other known *Flavobacterium* spp. and could be a target site for future fluorescent *in situ* hybridization probe or quantitative PCR (qPCR) assays. There is a growing recognition of the large number of opportunistic fish pathogens in the genus *Flavobacterium*, and these microorganisms have been implicated in both aquacultural and wild fish die-offs (38–40), which suggests that management of this genus is critical in recirculating aquaculture. However, control of *Flavobacterium* spp. is difficult since members of the genus often degrade and remineralize macromolecules and can subsequently survive outside the host (37, 41–43).

*Polynucleobacter* spp. and *Aurantimicrobium* spp. were most abundant in the aquaponic system samples but were absent or at very low relative abundances in all RAS samples. Both of these organisms are common members of freshwater lake/river microbial communities (44, 45). On the basis of this limited data set, it seems likely that aquaponic systems select for more-natural aquatic communities than those of RAS, presumably because of the availability of the plant-derived or phytoplankton-derived nutrients that are common in lakes/rivers and that would be available in these systems, e.g., glycolate for *Polynucleobacter* spp. (46). In the case of the Marinette facility, one tank was converted from an aquaponic system to a RAS. The aquaponic taxa were maintained at the same relative abundances in the RAS as they had been in the aquaponic setup (Fig. 3). This within-site community similarity despite changes in system design (i.e., RAS versus aquaponic) further supports our understanding that a facility’s overall conditions (e.g., source water, operator-controlled pH, temperature, light) and potential founder effects have a strong influence on the persistent microbiome. Despite the use of different taxonomic assignment procedures and different PCR primer sets, the data corresponding to taxon recovery from specific components and from particular system designs reported previously by Eck et al. (24) generally agree with our results. Additionally, those authors observed similar founder effects among their studied systems (25). As such, these results highlight the need to improve our understanding of processes that drive microbial community assembly in RAS and aquaponics and to determine whether control during start-up can improve long-term system performance/health.

### Nitrifier guilds across biological filters.

**FIG 4:**
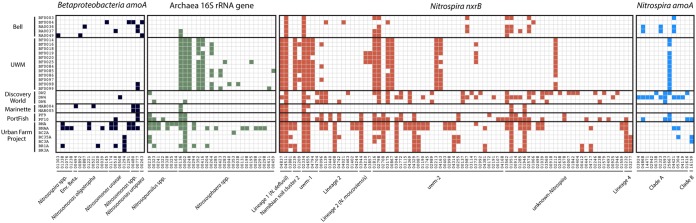
Presence/absence heat map of nitrifying microorganism amplicon sequence variants (ASVs). Samples are included as rows, and sample facilities are indicated. Columns represent unique ASVs (>95% nucleotide sequence identity) ordered by taxonomic affiliation as indicated. The presence of an ASV in a sample is indicated by a colored square. ASV designations are indicated along the *x* axis. Sequence alignments and phylogenies for *Betaproteobacteria amoA*, *Nitrospira nxrB*, and *Nitrospira amoA* ASVs can be found at https://doi.org/10.6084/m9.figshare.7777232.v1, https://doi.org/10.6084/m9.figshare.7777241.v2, and https://doi.org/10.6084/m9.figshare.7777247.v1.

Despite new discoveries in aquaculture nitrification/biofiltration (22, 35, 47–51), system designs often only consider the physiology and enzymatic rates of *Nitrosomonas* and *Nitrobacter* spp. (1, 52). Previously, *Nitrosomonas* sequence recovery was found to be influenced by a nutrient gradient and *Nitrobacter* were not recovered at all from a midsize RAS (22). Despite biogeographic differences in bacterial 16S rRNA gene community composition, certain nitrifying consortia were consistent across the systems surveyed. All sites had ASVs of ammonia-oxidizing archaea (AOA) (based on 16S rRNA gene sequence) and *Nitrospira* (based on *nxrB* gene sequence; Fig. 4). In fact, two *Nitrospira nxrB* sequences, one affiliated with lineage 1 *Nitrospira* (53) and another with the uwm-1 lineage (22), were identified in every sample collected (Fig. 4). The occurrence patterns of the AOA and *Nitrospira* genotypes were correlated across sites (Spearman’s rho Mantel test [ρ = 0.5073, *P* = 0.001]). We noted previously that AOA and *Nitrospira* genotype abundance patterns in a single RAS biological filter were correlated across a fish rearing cycle and that both groups of nitrifiers were present at >1 × 10^8^ nitrifying marker genes per gram of sand (22). AOA are favored over ammonia-oxidizing bacteria (AOB) under conditions of low ammonia substrate concentrations (54). Since ammonia oligotrophy is a design constraint in RAS and aquaponic systems, high abundances of AOA should be present in these environments. We found our results to be consistent with this idea, as AOA were the dominant ammonia-oxidizing taxa across nearly all RAS and aquaponic systems (Fig. 4). Going forward, AOA physiology rather than AOB physiology should form the basis of future nitrifying biofilter design and nitrogen flux modeling. It is unknown whether AOA arise as the dominant ammonia-oxidizing taxa in these systems at biofilter initiation or do so after substrate concentrations decrease and stabilize following the occurrences of the high ammonia concentrations typically seen during the start-up phase.

In this study, we found a diverse number of *Nitrospira amoA* genotypes (referred to here as complete-ammonia-oxidizing [“comammox”] genotypes), but nearly all were affiliated with the designated clade A type (Fig. 4; https://doi.org/10.6084/m9.figshare.7777241.v2). In contrast to this diversity, a single sequence was found in three of the four systems sourcing their water from Lake Michigan (no comammox were identified at the Marinette facility), but this sequence was not present in the systems with alternative water sources. Source water may be a major determinant of comammox association with aquaculture facilities. Municipal water sources seem to be a major habitat for comammox *Nitrospira*. Comammox have been found commonly in drinking water treatment plants (DWTPs) (55, 56). Comammox also have been enriched from potable water point of use (57), which could be their seed environment for many RAS or AP facilities.

Why comammox *Nitrospira* appear in these systems is still unclear, although one occurrence pattern was apparent. Comammox were present in the most oligotrophic systems that also contained fluidized sand filters (SFs). More data are needed to rigorously test this association. Rapid sand filters (RSF) used in drinking water treatment have been shown to harbor comammox (55, 56), as have RSFs processing groundwater (58). The commonality between DWTP RSF and freshwater RAS biofilters merits further study, as we have not yet ascertained an answer to the following question: do comammox *Nitrospira* spp. regularly associate with nitrifying biofilms formed on silica sand?

In samples containing comammox *amoA* amplicons, we found them to correlate with *nxrB* genotype occurrence patterns (Spearman’s rho Mantel test [ρ = 0.413, *P* = 0.001]), which supports the idea that only certain *nxrB* genotypes relate to comammox capabilities in the genus *Nitrospira* (22). On the basis of results from current kinetic experiments (59), it is likely that comammox *Nitrospira* species are competitive with AOA in RAS and aquaponic systems. If the competition between comammox and AOA were strong, the ammonia-oxidation niche within the biofilters would be mutually exclusive (i.e., the biofilters would harbor comammox or AOA but not both). We found that some facility biofilters contained both AOA and comammox, which may indicate that niche differentiation based on unknown traits and/or associations with other taxa are likely. It is also possible, given the current limited understanding of comammox *amoA* gene diversity, that the primers that we used did not fully capture the diversity of comammox *amoA* genes (60). In this scenario, comammox *Nitrospira* presence would be undercounted, thus obscuring their presence in a wide-range of facilities and a possible central role as ammonia oxidizers in all nitrifying biofilter RAS operations.

**FIG 5:**
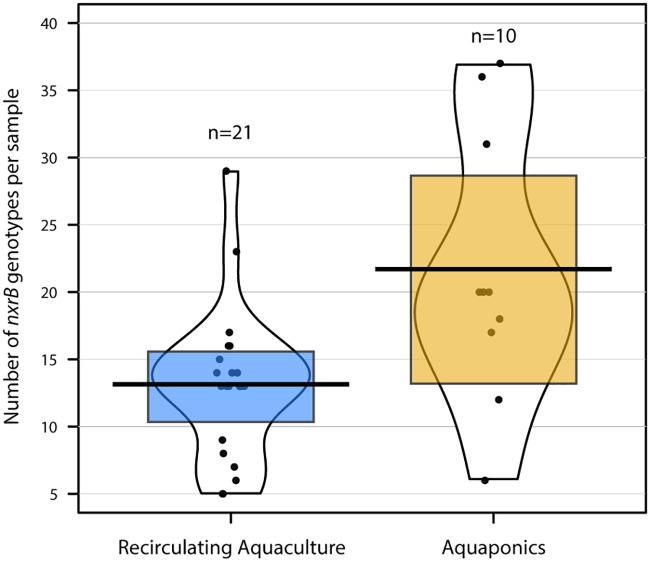
Richness distribution for recovered *nxrB* genotypes between RAS and aquaponic samples. Pirate plots (“yarrr” package in R) are indicated for *nxrB* genotype richness for each sample class (aquaponic or RAS). A black line indicates the richness mean, a colored box indicates the Bayesian highest-density interval, and all points are listed with corresponding indication of data distribution/density via figure shape.

Examination of *nxrB* genotypes as a proxy for nitrite-oxidation potential revealed that aquaponic facilities harbored significantly more genotypes of *Nitrospira nxrB* than RAS (Fig. 5; Kruskal-Wallis rank sum [comparing *nxrB* copy number to system type], χ^2^= 6.71, df = 1, *P* = 0.0096). Outside this system-level driver of diversity, there were no clear patterns corresponding to the presence of individual *nxrB* sequence variants across individual samples (Fig. 4). The recovered *nxrB* sequences were also spread widely across the *nxrB* phylogeny, including sequences from cryptic lineages as well as *Nitrospira* lineages I, II, and IV (https://doi.org/10.6084/m9.figshare.7777247.v1). The drivers of this phenomenon within *Nitrospira* diversity are not clear but could be related to source water variation or to priority effects during system colonization or could represent the result of increased variability in *nxrB* sequences between comammox and nitrite-oxidizing bacteria (NOB)–*Nitrospira* (61). We believe that this represents the first comparison of the levels of recovery of *Nitrospira nxrB* genotypes from aquaponic and RAS facilities; therefore, it is not clear whether this diversity should be expected. We hypothesize that increased *Nitrospira nxrB* diversity in aquaponic systems is a consequence of the increased trophic levels present, but this also remains to be tested. The high number of co-occurring *Nitrospira nxrB* gene variants within each facility also raises questions as to whether each of these presumed nitrite oxidizers contributes to nitrification and, if so, how these competing populations are maintained. Regardless, the mechanism of *Nitrospira* selection and optimization of the nitrifying capacity of various species/strains merit further study, as does the potential for nitrogen cycle niche substrate partitioning among closely related nitrifying microorganisms. Similarly to the *Nitrospira* data, there was a diverse set of AOA across systems, with multiple 16S rRNA gene ASVs co-occurring at most facilities (Fig. 4). Sequences classified as *Nitrososphaera* spp. were most prevalent across the systems (Fig. 4), but *Nitrosopumilus* spp. were also common.

It is clear that the low ammonia concentration within the RAS biofilters selects for AOA+*Nitrospira* mutualisms, as well as comammox *Nitrospira* spp. Although there appears to be significant diversity in the *Nitrospira* and AOA across RAS, particular *Nitrospira* and AOA gene variants tend to co-occur, which suggests that some mutualistic relationships are favored over others. Moreover, these results suggest that there is not a single ideal consortium of nitrifying microorganisms suitable for every system.

### Conclusions.

Despite differences in RAS operations and overall microbial community composition, all facilities retained some common (i.e., core) microorganisms associated with each of the major system components (rearing tank, solids clarifier, and biofilter). Facility, source water, and component association each influence RAS and aquaponic system microbial community composition. This suggests the potential for tractable study of trait-based microbial assemblages in RAS and aquaponics related to system operations. Additionally, these results offer some support for the decoupled aquaponic system model (18), since the beta diversity within a single system, and across systems, is coupled to component class. By decoupling components in RAS or aquaponic systems, one could avoid unwittingly designing a system’s “Achilles heel.” One of the systems surveyed had continual issues with solids clarifier failure, which in turn led to a suppression of nitrification and a spike in nitrite levels and to subsequent die-off of Perca flavescens due to outbreaks of the fish pathogen Flavobacterium columnare. Since, based on the results of this study, each component could be considered to represent its own microenvironment, decoupling components would allow aquaculturalists and aquaponics practitioners a greater level of system control.

Having conducted our survey of nitrogen cycle amplicon markers, it is apparent that the AOA-plus-NOB *Nitrospira* nitrifying guild is the most common across freshwater aquaria and RAS (22, 47, 62). It is also worth noting that, although the aquaculture practitioners from our survey were knowledgeable about nitrification as a system process, many believed that *Nitrosomonas* and *Nitrobacter* species were the sole nitrifying taxa present. The results from this study and others (63) indicate this is not the nitrification schema present in operational RAS, and it is our recommendation that aquacultural organizations incorporate new nitrogen cycle findings into stakeholder outreach plans to better inform system operators when they select starter cultures or substrates for a biological filter. Furthermore, our work and that of others (15, 19, 64) suggests that more “-omics” studies would benefit both aquaculture and aquaponic system development. Subtle differences in microbial assemblages may impart significantly different health, production, and operations outcomes beyond what is traditionally known. Ultimately, we believe that identifying key microorganisms for RAS and then deciphering their roles will enable targeted controls to increase fish and plant yields.

## MATERIALS AND METHODS

### Sample collection, processing, and DNA extraction.

**FIG 6:**
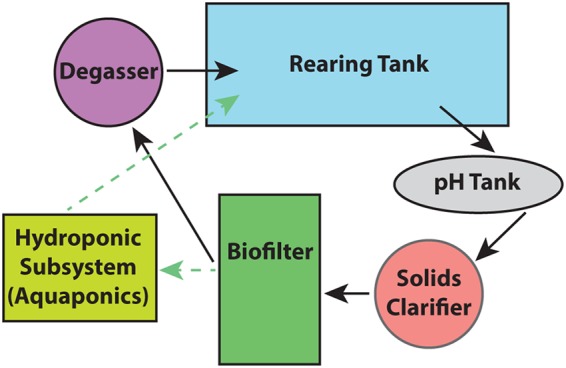
Generalized component process diagram. Black arrows indicate the flow of the water through the University of Wisconsin—Milwaukee (UWM) system. The dashed green arrows represent the generalized flow of water in the aquaponic systems.

We collected samples from the UWM SFS RAS components (rearing tank, pH tank, solids clarifier, biofilter, and degasser) over a period of 7 months. A generalized diagram of components sampled across all systems is shown in Fig. 6. We also collected samples from two additional aquaculture facilities (Marinette and Bell Aquaculture), from three aquaponic systems growing lettuce varietals (Marinette, PortFish, and the Urban Farm Project), and from a set of recirculating freshwater aquaria (Discovery World, Milwaukee, WI). No commercial starter cultures were used to initiate any system. Further system setup details, sample location details, and water quality metadata associated with these facilities are listed in Table S1 in the supplemental material. All samples were collected using autoclaved 500-ml plastic bottles. All water samples were filtered using 0.22-μm-pore-size filters (EMD Millipore, Darmstadt, Germany) (47-mm-diameter mixed-cellulose esters) and frozen at −80°C until further processing was performed. The filtered volume for each sample is listed in Table S1. Where applicable, biofilter pore water samples were collected and decanted from the biofilter solid medium substrate, and ∼1 g (wet weight) of the remaining substrate was frozen at −80°C until extraction. Sample sites, available operator data, and weights or volumes of samples extracted are listed in Table S1.

10.1128/mSphere.00143-19.1 TABLE S1 Sample list and associated metadata for the V6 and V4-V5 16S rRNA gene sequence datasets. Download Table S1, XLSX file, 0.1 MB. Copyright © 2019 Bartelme et al. 2019 Bartelme et al. This content is distributed under the terms of the Creative Commons Attribution 4.0 International license.

Prior to DNA extraction, sample filters were removed from the freezer and macerated with a sterilized spatula. DNA was then extracted using an MP Bio FastDNA spin kit for soil (MP Bio, Solon, OH, USA) according to the manufacturer's instructions except that each sample underwent 2 min of bead beating using the beads included in the MP Bio FastDNA spin kit and a Mini-BeadBeater-16 at the units’ fixed speed (Biospec Products, Inc., Bartlesville, OK, USA). The initial quality of extracts was assessed using a NanoDrop Lite spectrophotometer (Thermo Fisher Scientific Inc., Waltham, MA, USA).

### High-throughput sequencing reactions.

Two different Illumina platforms were utilized for massively parallel paired-end sequencing of bacterial 16S rRNA gene amplicons. For the within-system component comparison, we targeted the V6 region of the 16S rRNA gene (65). We used 5 to 20 ng of the UWM SFS RAS component DNA extracts in a reaction mixture consisting of 4 units of Invitrogen Platinum HiFi *Taq* polymerase, 2 mM MgSO_4_, 0.2 mM Invitrogen dNTPs, and 0.2 μM combined primers (Table 2) at a volume of 100 μl. These master mix reaction mixtures were split in triplicate, amplified with PCR, cleaned, etc., as described in reference 65. Barcoded amplicon libraries were generated and sequenced on an Illumina HiSeq system at the Marine Biological Laboratory (MBL) in Woods Hole, MA. For the cross-site comparisons, the V4-V5 region of the 16S rRNA gene was targeted. Each sample was PCR amplified in triplicate using three separate Eppendorf Mastercycler Pro thermocyclers (Eppendorf, Mt. Laurel, NJ, USA) and previously published primers (Table 2) purchased from IDT (Integrated DNA Technologies, Coralville, IA, USA). All PCR products were cleaned using Ampure beads (Beckman Coulter, Inc., Brea, CA, USA) prior to library preparation, and the resultant DNA quality and the concentrations of all samples were checked using the BroadRange Qubit 2.0 spectrophotometric assay (Thermo Fisher Scientific Inc., Waltham, MA, USA). MiSeq sequencing was carried out either at the Great Lakes Genomic Center (Milwaukee, WI, USA) or at the MBL (Woods Hole, MA, USA).

**TABLE 2:** PCR primers used in this study

Gene target	Forward primer(s) (5′–3′)	Reverse primer(s) (5′–3′)	Component(s) surveyed	Sample site(s)	Reference(s)
Bacterial 16S rRNA gene V6 region	CTAACCGANGAACCTYACC, CNACGCGAAGAACCTTANC, CAACGCGMARAACCTTACC, ATACGCGARGAACCTTACC	CGACRRCCATGCANCACCT	All	UWM RAS	72
Bacterial 16S rRNA gene V4-V5 region	CCAGCAGCYGCGGTAAN	CCGTCAATTCNTTTRAGT, CCGTCAATTTCTTTGAGT, CCGTCTATTCCTTTGANT	All	All	73
Archaeal 16S rRNA gene V4-V5 region	GCCTAAAGCATCCGTAGC, GCCTAAARCGTYCGTAGC, GTCTAAAGGGTCYGTAGC, GCTTAAAGNGTYCGTAGC, GTCTAAARCGYYCGTAGC	CCGGCGTTGANTCCAATT	Biofilters	All biofilter samples	74
Betaproteobacterial *amoA*	GGGGHTTYTACTGGTGGT	CCCCTCKGSAAAGCCTTCTTC	Biofilters	All Biofilter Samples	75, 76
Comammox *amoA*	GGAYTTYTGGNTNGATTGGA	WRKTNNGACCACCASKACCA	Biofilters	All biofilter samples	Modified from reference 58
*Nitrospira nxrB*	TACATGTGGTGGAACA	CGGTTCTGGTCRATCA	Biofilters	All biofilter samples	53

### Bacterial rRNA gene sequence data processing.

All bacterial 16S rRNA gene sequences were trimmed of their respective primers using the Great Lakes Genomic Center GNU parallel implementation of CutAdapt (66). After primer trimming, reads were merged with PEAR (67). For the V6 data, paired reads with mismatches were removed from further analysis. For the V4-V5 data, we allowed 1 mismatch between the reads and used the nucleotide call with the higher quality score in the final merged sequence. The PEAR output was converted from FASTQ format to FASTA using the FASTX Toolkit. After merging and trimming, the V6 and V4-V5 16S rRNA gene data sets were decomposed into representative minimum entropy decomposition (MED) nodes (equivalent to operational taxonomic units [OTUs]/amplicon sequence variants [ASVs]) with default settings except that the respective minimum substantive abundance cutoffs were set to 330 and 398, respectively. Chimera checking was carried out against the SILVA gold reference database with the implementations of Chimera Slayer and Uchime in mothur (68). Chimeric node sequences were removed from the FASTA and absolute abundance tables generated by MED before taxonomy or statistical calculation. Taxonomy was assigned to nonchimeric MED nodes using the SILVA 128 SSU database and SINA online (69). FASTA files of representative nodes exceeding the SINA sequence number limit were split using the Great Lakes Genomic Center’s SplitFA program. MED nodes not matching known bacterial taxonomies were removed from the MED node absolute-counts table and eliminated from downstream statistical analyses. See Table S1 for raw and processed read counts.

### Within-system diversity calculations and statistical tests.

Alpha and beta diversity comparisons were used to test influences on component bacterial community composition pertaining to the RAS environment and resultant environmental influences on bacterial taxonomic abundance. Alpha diversity was calculated using the natural logarithm base Shannon-Weaver Index (*H*′) from the vegan R package diversity function (70). MED node evenness (Pielou’s *J*) was derived in R, where *J* = *H*′/log(*S*) and *S* were calculated using the specnumber function and the relative-abundance table (70). Kruskal-Wallis rank sum analysis was then utilized for hypothesis testing of the influence of sample type (planktonic, sludge, or biofilm) on alpha diversity.

The chosen beta diversity metric, Bray-Curtis dissimilarity, was calculated using the vegdist function from vegan across the UWM SFS RAS V6 16S rRNA gene data set (70). ADONIS was then used to test the hypothesis that Bray-Curtis dissimilarity would reflect the association with each component as its own environment with significantly different relative taxonomic abundances (70). The ADONIS function was run with 999 permutations, where Bray-Curtis was the dependent variable and component association was the independent variable. Component association was defined as represented by a sample originating from the interstitial water, biofilm sand, solid sludge, or effluent of a particular RAS component (rearing tank, pH tank, solids clarifier, biofilter, or degasser).

### Cross-system analyses, ordination, and shared taxonomic calculations.

Samples were collected from six aquaculture and aquaponic facilities to generate the V4-V5 16S rRNA gene data used in this cross-system comparison (Table S1). The system component classes (rearing tank, pH tank, solids clarifier, biofilter, and degasser) for calculating facility diversity metrics were extended to include hydroponic subsystem samples from aquaponic facilities and conditioning water samples. The V4-V5 MED node relative-abundance table was used as input for nMDS (70) with vegan’s metaMDS function, using *k *=* *5 dimensions and a Bray-Curtis dissimilarity matrix.

### Nitrification marker gene amplification, multiplex reaction, and analysis.

A multiplex MiSeq assay was constructed that targeted the following nitrification marker genes: *amoA* from *Betaproteobacteria*, *amoA* of complete-ammonia-oxidizing *Nitrospira*, *nxrB* from *Nitrospira*, and the V4-V5 region of the *Archaea* 16S rRNA gene for ammonia-oxidizing archaea (Table 2). Only samples associated with biological filtration were used as templates for the multiplex assay. Briefly, copies of the primers listed in Table 2 were ordered with Illumina TruSeq adapter sequences from IDT (Integrated DNA Technologies, Coralville, IA, USA). Each 20-μl reaction mixture consisted of HiFi master mix (Kapa Biosystems) (2×, 10 μl) and 200 nM (final concentration) forward and reverse primers with 10 to 100 ng of sample genomic DNA (gDNA). PCR products were amplified in triplicate across three separate Eppendorf Mastercycler Pro thermocyclers at the Great Lakes Genomics Center. Triplicate products were pooled by gene target and were cleaned with Ampure XP beads according to the instructions of the manufacturer. Template concentrations were quantified with QuantIT PicoGreen (Thermo Fisher). After quantification, amplicons from the four PCR assays were pooled in a volume of 40 μl at an approximate concentration of 1.8 ng/μl (betaproteobacterial *amoA*, *Nitrospira nxrB*, and *Archaea* 16S rRNA gene V4-V5), while 0.9 ng/μl of *Nitrospira amoA* products were used to account for the shorter product length. After pooling, each well was barcoded by sample using Nextera Adapter sequences.

Amplicons were demultiplexed from the MiSeq sequencer using the Nextera tags and were then merged and further demultiplexed by the use of target genes with mothur (68). Sequences were decomposed into unique ASVs using MED (71), with minimum substantive abundances (-M) set for each gene according to best practices outlined elsewhere (http://merenlab.org/2013/11/04/oligotyping-best-practices/). To further denoise the amplicon data, opticlust was implemented within mothur to cluster MED node ASVs at 95% nucleotide sequence identity for betaproteobacterial *amoA*, *Nitrospira nxrB*, and complete-ammonia-oxidizing (comammox) *Nitrospira amoA*. After representative sequences were identified for each sequence cluster (i.e., the ≥95% clusters for the protein-encoding genes or the MED nodes for the 16S rRNA genes), taxonomy was assigned via two methods, and *Archaea* V4-V5 16S rRNA gene identity was assigned using SINA and version 128 of the SILVA database (69). Any unknown sequences were removed before further analysis. For betaproteobacterial *amoA*, comammox *amoA*, and *Nitrospira nxrB*, reads were aligned to the ARB databases (described previously in reference 22) (see https://doi.org/10.6084/m9.figshare.7777232.v1, https://doi.org/10.6084/m9.figshare.7777241.v2, and https://doi.org/10.6084/m9.figshare.7777247.v1 for the ARB databases and included alignments). Sequences falling outside (i.e., basal to) the known marker gene diversity in phylogenetic reconstructions were compared against the NCBI nucleotide database using blastn on default settings. Those nitrification marker sequences that matched only a small portion of a known corresponding gene or no known nitrification target gene sequences were assumed to be chimeras or to represent nonspecific amplification and were removed from downstream analyses.

In order to reduce levels of sequencing errors derived from the Illumina MiSeq sequencing procedure, all ASVs of the four genes with absolute abundances of <10 in any single sample were assumed to represent noise, lane drift, or chimeras. These abundances were converted to zero within R before further analysis was performed. Nonamplification of a gene in a sample was also assumed to be equivalent to an absolute abundance of zero. After the data tables were finalized in R, binary Jaccard dissimilarities were calculated for each gene using vegan (70). To test correlations between the binary dissimilarity matrices, Spearman’s ρ Mantel tests were conducted for 999 iterations for all possible pairwise combinations of the four Jaccard dissimilarities.

### Data availability.

Data are available on the NCBI sequence read archive with the following accession numbers: for bacterial V6 16S rRNA gene data, accession no. SRP162340; for bacterial V4-V5 16S rRNA gene data, accession no. SRP162354; and for the multiplexed nitrification marker gene data, accession no. SRP162338.
